# Time-dependent variation of lead isotopes of lead white in 17th century Dutch paintings

**DOI:** 10.1126/sciadv.abi5905

**Published:** 2021-12-01

**Authors:** Paolo D’Imporzano, Katrien Keune, Janne M. Koornneef, Erma Hermens, Petria Noble, A. L. S. Vandivere, Gareth R. Davies

**Affiliations:** 1 Faculty of Science, Vrije University Amsterdam, Amsterdam, Netherlands.; 2 Conservation & Science, Rijksmuseum, Amsterdam, Netherlands.; 3 Faculty of Science, University of Amsterdam, Amsterdam, Netherlands.; 4 Faculty of Humanities, University of Amsterdam, Amsterdam, Netherlands.; 5 Conservation Department, Mauritshuis, The Hague, Netherlands.

## Abstract

This study investigates how lead isotopes in lead white pigment can be used as an additional diagnostic tool to constrain the production time of 17th century Dutch paintings. Analysis of 77 well-dated paintings from 27 different Dutch artists reveal significant change in the source of lead used in lead white at the start, middle, and end of the 17th century. Isotopic shifts are related to major historical and socioeconomical events such as the English Civil War and Anglo-Dutch-French conflicts. These observations offer the prospect that lead isotope analysis of lead white could aid attribution and authentication of Dutch 17th century paintings and provide insights into artists’ international travels as well as lead production and trading.

## Teaser

Changing lead isotope ratios of 17th century Dutch paintings over time suggest usefulness of isotope data for art authentication.

## INTRODUCTION

Lead isotope analysis has been widely applied in the field of cultural heritage since the 1960s (*1*–*4*). Applications have addressed the provenance of metal artefacts produced by civilizations active in prehistoric, Greek, Roman, and up to early medieval times. In all these cases, the concept is to use the lead (Pb) isotopes to fingerprint the possible geological source metal. Keisch and Callahan (*5*) reported the first application of lead isotope analysis to lead white pigment. Since that time, the method has been used to examine differences in pigment production, both in terms of the control of physical pigment properties and the possibility to determine regions of manufacture and use establishing, for example, clear isotopic difference between lead white produced in northern and southern Europe (*5*–*8*). The current study investigates the possibility of using lead isotope analysis in 17th century Netherlands paintings to determine the time of painting production.

Lead white is a pigment that was used in paintings from antiquity until the 20th century and is considered the most important of all white pigments (*9*). When the white powder is mixed with a binder, such as linseed oil, it creates a versatile paint. It was used pure, for instance, for white highlights or mixed with other pigments to obtain lighter tones. It can also be a component of the ground layer (*9*). The pigment itself can be used as a siccative agent to speed up the drying of oil paint. Lead white is a lead carbonate composed of a mixture of two main phases: cerussite (PbCO_3_) and hydrocerussite [2PbCO_3_·Pb(OH)_2_] (*10*), but it can also contain a low percentage of other lead salts such as plumbonacrite [Pb_5_O(OH)_2_(CO_3_)_3_] (*11*–*13*). Lead represents between 77 and 80% by weight of the lead white. The high content of Pb in the pigment, combined with its ubiquitous presence in oil paintings until the 20th century, makes lead white the perfect candidate for lead isotope analysis. Lead isotope analysis of lead white can help determine the geographic origin of the lead ore. This information can be useful for tracing the trade routes of lead and provide information about the history of the pigment.

In the last decades, lead isotope analysis of lead white (*6*–*8*, *14*, *15*) focused on the following: (i) the place of origin of the lead ore, i.e., its provenance (*5*, *7*, *8*); (ii) identification of paintings according to artistic groups (*7*, *8*, *15*); (iii) investigating the composition/variation of lead isotope of lead white within a single painting (*6*); and (iv) authentication of paintings, as in the case of the attribution of a copy of *Saint Praxedis* to Vermeer (*16*, *17*) or the attribution of Michiel Sweerts paintings to his period in Rome (*18*). Most recently, lead isotope analysis of lead white has also been coupled with the study of ^14^C of the carbonate for dating purposes (*15*, *19*).

This study evaluates the potential use of lead isotope analysis on lead white samples from well-dated (± 2.5 years ca.) paintings produced in The Netherlands in the 17th century. Lead white was sampled from 77 authenticated paintings, with known date of production, from Dutch museums (Rijksmuseum, Amsterdam and Mauritshuis, The Hague) by 27 artists who were active in the 17th century. On the basis of the variation in lead isotope ratios, time-dependent clusters can be identified.

The study establishes the basis for an international lead isotope database of lead white, which will provide a better understanding of the historical use of lead white across Europe. The database can be used to gain information about working practices of the artists and their travels. Moreover, by linking the lead isotope composition of lead white to a specific historical period, isotope analysis can be an additional tool for the attribution of disputed paintings.

Lead isotope analysis is based on the study of the four stable isotopes of lead: ^204^Pb, ^206^Pb, ^207^Pb, and ^208^Pb. ^204^Pb is nonradiogenic, while ^206^Pb, ^207^Pb, and ^208^Pb are the product of radioactive decay of ^238^U, ^235^U, and ^232^Th, respectively. The long duration of geological activity, more than 4.5 billion years, coupled with variations in U/Pb and Th/Pb in different geological settings on Earth, means that worldwide ore deposits were formed billions of years apart and involved sources of metals with distinct Pb isotope ratios. Consequently, galenas from ore deposits are characterized by distinct lead isotope compositions that reflect the distinct time of formation and the different U/Pb and Th/Pb ratios of the metal source (distinct time-integrated U/Pb and Th/Pb) (*20*–*22*). When extracted by smelting, the lead present in these deposits retains the isotopic composition of the original ore minerals, and therefore, it can be possible to use lead isotope ratios to identify the provenance of lead-rich artefacts, alloys, and pigments (*1*, *2*, *5*–*8*). This characteristic, in the context of lead white, also allows the identification of specific artistic groups that were active in different regions where different lead sources were used to produce the pigment. This is evident from the markedly different lead isotope compositions of lead white used by 16th to 17th century artists from Italy (south of the Alps) and The Netherlands (north of the Alps) (*6*–*8*).

To correctly interpret the lead isotope data of a painting, detailed knowledge of the raw materials involved in the production of lead white is required, including trading practices and historical events that may have affected its production. To study the variation of lead isotope ratios in 17th century Dutch paintings, the main producers of lead and lead white who traded in The Netherlands must be identified. The Netherlands, or the United Provinces as it was then known, was the main production center of lead white pigment at the time and was able to meet the internal demand for this product. Although the exact production of lead white during the 17th century is not known precisely, it is estimated that in 1790, about 1350 tons of lead white were shipped from the harbors of Amsterdam and Rotterdam and that at the end of the 18th century, more than 35 factories were active in The Netherlands with a production of 4000 tons of lead white per year (*23*, *24*). In the 17th century, other smaller production centers were active in England and Venice, the latter being the main lead white producer from the middle ages until the 17th century (*23*–*25*).

The Netherlands imported metallic lead to make lead white from abroad, and it was usually melted and recast as thin coils. This implies that there was recycling/mixing of different leads with different isotopic compositions, which can be identified by lead isotope analysis. Written records (*26*–*28**)* and lead isotope studies (*6*–*8*) indicate that England was the main lead source in Europe in the 17th century and that lead white produced in The Netherlands was most likely made using English lead.

The lead white production system used in The Netherlands in the 17th century is known as the “Dutch stack process” and is described in detail by Homburg *et al*. (*23*). It mainly consists of the stacking of several pots, containing vinegar and metallic lead coils (metal and liquid are not in contact), and covering them with horse manure. The acid vapors of the vinegar together with the CO_2_ and heat produced by the manure accelerated reaction of the metallic lead to form lead carbonate (lead white) in a process that lasted several weeks. The pigment was separated from the unreacted metal, which is recycled, while the lead white is washed and transformed to the final product (*24*, *28*). The fact that the lead was recycled, and probably at a certain moment supplemented with a new batch of lead, opens up a series of questions on the variability of lead isotopes of lead white that will be examined in this study.

This study combines the date and location of painting production with lead isotope ratios determined in lead white from the painting, and places the data in the context of historical socioeconomical events. This integrated approach will contribute new and increasingly more accurate information that can be used by cultural heritage researchers to date, attribute, and understand artworks.

## RESULTS

Lead white was sampled from paintings that cover a time period from 1588 to 1700, with three paintings from the end of the 16th century (Table 1 and table S1). The majority of the paintings were made in The Netherlands, but four samples came from paintings dated to periods in which the Dutch artists travel outside the country. They will be discussed in detail below. The lead isotope data for lead whites in 17th century Dutch paintings are generally comparable with the available literature data (*7*, *8*, *26*, *29*). Overall, the lead isotope ratios of the lead white range from 18.386 to 18.524 for ^206^Pb/^204^Pb, 15.625 to 15.642 for ^207^Pb/^204^Pb, and from 38.372 to 38.499 for ^208^Pb/^204^Pb.

**Table 1.** Paintings sorted according to artist. For each painting, the following are reported: sample museum identification (ID), lead isotope ratios with 2SE, year of production (±2.5 years), and calculated LIRI.

**Artist**	**Museum ID**	**^206^Pb/^204^Pb**	**2SE**	**^207^Pb/^204^Pb**	**2SE**	**^208^Pb/^204^Pb**	**2SE**	**^207^Pb/^206^Pb**	**2SE**	**^208^Pb/^206^Pb**	**2SE**	**Year**	**LIRI**
**Alewijn A.**	SK-A-1726	18.4321	0.0005	15.6305	0.0009	38.449	0.008	0.84801	0.00005	2.08595	0.00005	1700	18.25
**Anonymous**	SK-A-1312	18.4677	0.0005	15.6367	0.0009	38.439	0.001	0.84671	0.00004	2.08138	0.00003	1631	18.30
**Berchem N. P.**	SK-A-27	18.4806	0.0004	15.6335	0.0007	38.448	0.001	0.84594	0.00003	2.08043	0.00002	1647	18.32
**Berchem N. P.**	SK-A-32	18.4788	0.0010	15.6371	0.0018	38.444	0.010	0.84622	0.00003	2.08043	0.00006	1645	18.32
**Berchem N. P.**	SK-A-28	18.4867	0.0005	15.6396	0.0005	38.459	0.003	0.84599	0.00002	2.08035	0.00006	1647	18.32
**Berchem N. P.**	SK-A-31	18.3863	0.0004	15.6267	0.0007	38.372	0.001	0.84991	0.00003	2.08697	0.00002	1655	18.23
**Berchem N. P.**	SK-A-29	18.4739	0.0004	15.6400	0.0006	38.444	0.001	0.84660	0.00002	2.08098	0.00002	1656	18.31
**Berchem N. P.**	SK-A-30	18.4846	0.0005	15.6381	0.0008	38.452	0.006	0.84600	0.00001	2.08019	0.00007	1656	18.32
**Berchem N. P.**	SK-A-680	18.4677	0.0016	15.6360	0.0017	38.429	0.008	0.84667	0.00004	2.08088	0.00006	1658	18.31
**Bol F.**	SK-A-714	18.4733	0.0005	15.6362	0.0001	38.439	0.000	0.84642	0.00002	2.08078	0.00004	1643	18.31
**Bol F.**	SK-A-613	18.4844	0.0005	15.6387	0.0006	38.449	0.001	0.84605	0.00002	2.08004	0.00002	1664	18.32
**Bol F.**	SK-A-614	18.4730	0.0021	15.6315	0.0004	38.456	0.009	0.84618	0.00012	2.08172	0.00002	1664	18.31
**Bol F.**	SK-A-45	18.4801	0.0015	15.6367	0.0026	38.453	0.004	0.84614	0.00004	2.08075	0.00004	1666	18.32
**Borch G. ter**	SK-A-1784	18.4571	0.0114	15.6310	0.0050	38.417	0.027	0.84688	0.00003	2.08141	0.00002	1644	18.30
**Borch G. ter**	SK-A-3842	18.4672	0.0015	15.6356	0.0007	38.437	0.001	0.84667	0.00003	2.08134	0.00002	1647	18.31
**Borch G. ter**	SK-A-1786	18.4760	0.0065	15.6372	0.0030	38.442	0.009	0.84635	0.00004	2.08061	0.00002	1648	18.31
**Borch G. ter**	SK-A-4039	18.4763	0.0005	15.6370	0.0006	38.445	0.001	0.84633	0.00002	2.08077	0.00002	1652	18.31
**Borch G. ter**	SK-A-4038	18.4633	0.0014	15.6333	0.0006	38.427	0.001	0.84672	0.00002	2.08121	0.00002	1655	18.31
**Borch G. ter**	SK-A-2417	18.5168	0.0013	15.6310	0.0022	38.492	0.013	0.84415	0.00005	2.07873	0.00007	1670	18.35
**Brouwer A.**	MH919 2a	18.4410	0.0005	15.6289	0.0005	38.408	0.001	0.84751	0.00001	2.08273	0.00002	1625	18.28
**Brouwer A.**	MH067x2	18.4519	0.0007	15.6337	0.0006	38.425	0.001	0.84727	0.00001	2.08243	0.00002	1635	18.29
**Coques**	238X18 9/01MW	18.4765	0.0021	15.6376	0.0005	38.451	0.009	0.84635	0.00004	2.08104	0.00012	1670	18.31
**Drost W.**	SK-C-1802	18.4699	0.0015	15.6413	0.0005	38.450	0.002	0.84685	0.00008	2.08174	0.00002	1656	18.30
**Gheyn (II) J. de**	SK-A-2395	18.4434	0.0009	15.6294	0.0007	38.415	0.001	0.84742	0.00003	2.08285	0.00002	1607	18.28
**Grueber J. F.**	SK-A-2564	18.4758	0.0007	15.6382	0.0018	38.448	0.002	0.84642	0.00002	2.08097	0.00002	1670	18.31
**Hals F.**	459*10a06	18.4503	0.0002	15.6312	0.0000	38.424	0.000	0.84721	0.00000	2.08256	0.00003	1625	18.29
**Helst B. vd**	SK-A-147	18.4720	0.0004	15.6356	0.0006	38.442	0.001	0.84645	0.00002	2.08109	0.00002	1642	18.31
**Helst B. vd**	SK-A-142	18.4754	0.0017	15.6357	0.0001	38.444	0.001	0.84630	0.00001	2.08078	0.00004	1652	18.31
**Isaacsz P.**	SK-C-455	18.4484	0.0012	15.6353	0.0011	38.425	0.005	0.84752	0.00001	2.08284	0.00001	1599	18.28
**Maes**	MH0718X01	18.4729	0.0017	15.6324	0.0013	38.460	0.010	0.84624	0.00007	2.08197	0.00001	1682	18.30
Mierevelt M. J. Van*	SK-C-1481	18.4497	0.0018	15.6327	0.0031	38.431	0.008	0.84700	0.00008	2.08219	0.00002	1633	18.29
**Neer vd**	862X01 2011 SM	18.4722	0.0010	15.6362	0.0003	38.451	0.009	0.84647	0.00048	2.08153	0.00004	1675	18.31
**Ostade A. van**	SK-A-4093	18.4671	0.0004	15.6383	0.0008	38.450	0.001	0.84682	0.00003	2.08203	0.00002	1645	18.30
**Ostade A. van**	SK-A-300	18.4759	0.0005	15.6368	0.0006	38.444	0.001	0.84634	0.00002	2.08076	0.00001	1648	18.31
**Ostade A. van**	SK-A-3281	18.4736	0.0004	15.6368	0.0006	38.441	0.001	0.84644	0.00002	2.08081	0.00002	1665	18.31
**Ostade A. van**	SK-A-299	18.4672	0.0004	15.6345	0.0006	38.451	0.001	0.84661	0.00003	2.08211	0.00002	1671	18.30
**Ostade A. van**	SK-A-298	18.3957	0.0004	15.6390	0.0006	38.409	0.001	0.85015	0.00003	2.08793	0.00001	1673	18.22
**Palin M.^†^**	SK-A-3766	18.4744	0.0005	15.6325	0.0008	38.435	0.001	0.84617	0.00003	2.08042	0.00002	1690	18.32
**Palin M.^†^**	SK-A-3767	18.4813	0.0005	15.6331	0.0008	38.469	0.001	0.84589	0.00003	2.08148	0.00002	1690	18.31
**Pietersz (I) P.**	SK-A-3864	18.4676	0.0004	15.6326	0.0005	38.451	0.001	0.84649	0.00002	2.08207	0.00002	1588	18.30
**Pietersz (I) P.**	SK-A-3865	18.4635	0.0005	15.6267	0.0006	38.437	0.001	0.84636	0.00003	2.08178	0.00002	1588	18.30
**Post F. J.**	SK-A-4271	18.4483	0.0016	15.6325	0.0011	38.437	0.004	0.84737	0.00002	2.08348	0.00002	1637	18.28
**Post F. J.**	SK-A-3224	18.4763	0.0004	15.6396	0.0007	38.454	0.001	0.84647	0.00003	2.08124	0.00002	1652	18.31
**Post F. J.**	SK-A-1486	18.4799	0.0004	15.6424	0.0007	38.457	0.001	0.84646	0.00003	2.08099	0.00002	1659	18.31
**Post F. J.**	SK-A-742	18.4835	0.0004	15.6369	0.0008	38.441	0.001	0.84599	0.00003	2.07971	0.00002	1662	18.33
**Post F. J.**	SK-A-2333	18.4795	0.0004	15.6350	0.0008	38.462	0.001	0.84607	0.00003	2.08133	0.00002	1675	18.31
**Post F. J.**	SK-A-2334	18.4742	0.0004	15.6381	0.0006	38.458	0.001	0.84648	0.00002	2.08166	0.00002	1675	18.30
**Post F. J.**	SK-A-4272	18.4993	0.0007	15.6343	0.0017	38.494	0.004	0.84513	0.00006	2.08079	0.00001	1677	18.33
**Post F. J.**	SK-A-4273	18.4777	0.0014	15.6358	0.0002	38.481	0.007	0.84620	0.00002	2.08251	0.00009	1677	18.30
**Ravesteven**	421-2-CE	18.4638	0.0009	15.6378	0.0008	38.442	0.002	0.84695	0.00001	2.08199	0.00002	1612	18.30
**Ravesteven**	142/08/98	18.4480	0.0009	15.6394	0.0008	38.435	0.002	0.84776	0.00001	2.08338	0.00002	1616	18.28
**Rembrandt**	SK-A4717	18.4525	0.0004	15.6321	0.0007	38.430	0.001	0.84715	0.00003	2.08261	0.00002	1626	18.29
**Rembrandt**	SK-A-4691	18.4615	0.0004	15.6326	0.0006	38.436	0.001	0.84677	0.00002	2.08192	0.00001	1628	18.30
**Rembrandt**	148/3/98	18.4364	0.0010	15.6333	0.0008	38.415	0.002	0.84796	0.00000	2.08364	0.00001	1629	18.27
**Rembrandt**	MH0565x08	18.4475	0.0008	15.6369	0.0006	38.422	0.002	0.84764	0.00001	2.08277	0.00002	1630	18.28
**Rembrandt**	SK-A4833	18.4453	0.0025	15.6267	0.0024	38.432	0.014	0.84719	0.00005	2.08352	0.00007	1634	18.28
**Rembrandt**	SK-A-1935	18.4455	0.0004	15.6357	0.0007	38.422	0.001	0.84767	0.00003	2.08295	0.00002	1639	18.28
**Rembrandt**	SK-A-91	18.4625	0.0004	15.6339	0.0006	38.445	0.001	0.84679	0.00002	2.08229	0.00002	1642	18.30
**Rembrandt**	SK-A-4119	18.4669	0.0009	15.6369	0.0007	38.440	0.002	0.84675	0.00001	2.08152	0.00001	1645	18.32
**Rembrandt**	SK-A-4119	18.4669	0.0009	15.6369	0.0007	38.440	0.002	0.84675	0.00001	2.08152	0.00001	1645	18.30
**Rembrandt**	560-3b-CE98	18.4806	0.0010	15.6388	0.0008	38.456	0.002	0.84623	0.00001	2.08087	0.00001	1650	18.31
**Rembrandt**	AK-A-4050	18.4779	0.0004	15.6362	0.0006	38.443	0.001	0.84621	0.00002	2.08044	0.00001	1661	18.32
**Rijck P. C. van^†^**	SK-A-868	18.4591	0.0022	15.6313	0.0016	38.427	0.006	0.84681	0.00001	2.08168	0.00008	1615	18.30
**Rubens**	252/1098	18.4704	0.0009	15.6401	0.0008	38.444	0.002	0.84677	0.00000	2.08135	0.00001	1616	18.30
**Rubens**	926*05′5BS	18.4613	0.0010	15.6337	0.0008	38.426	0.002	0.84683	0.00001	2.08140	0.00002	1623	18.30
**Santvoort D. D. van**	SK-A-365	18.4639	0.0004	15.6324	0.0006	38.439	0.001	0.84665	0.00002	2.08182	0.00002	1635	18.30
**Santvoort D. D. van**	SK-A-1318	18.4488	0.0041	15.6309	0.0040	38.425	0.007	0.84726	0.00002	2.08275	0.00003	1638	18.29
**Santvoort D. D. van**	SK-A-1310	18.4636	0.0005	15.6334	0.0006	38.434	0.001	0.84672	0.00002	2.08157	0.00002	1644	18.30
**Santvoort D. D. van**	SK-A-1311	18.4449	0.0004	15.6369	0.0006	38.420	0.001	0.84776	0.00002	2.08293	0.00002	1644	18.28
**Santvoort D. D. van**	SK-A-1623	18.4741	0.0004	15.6338	0.0006	38.445	0.001	0.84626	0.00002	2.08098	0.00002	1650	18.31
**Schalcken**	160X2 1.11CP	18.4859	0.0035	15.6251	0.0019	38.460	0.009	0.84662	0.00002	2.08388	0.00003	1690	18.28
**Steen J.**	MH0165x02	18.4754	0.0006	15.6396	0.0005	38.447	0.001	0.84651	0.00001	2.08097	0.00002	1651	18.31
**Steen J.**	Brawn 056x02	18.4882	0.0009	15.6338	0.0008	38.464	0.002	0.84561	0.00001	2.08041	0.00001	1653	18.32
**Steen J.**	MH0166x05	18.4821	0.0010	15.6368	0.0008	38.445	0.002	0.84605	0.00000	2.08012	0.00001	1660	18.32
**Steen J.**	B143x01	18.4858	0.0006	15.6403	0.0005	38.459	0.001	0.84607	0.00000	2.08045	0.00001	1661	18.31
**Steen J.**	Brawn363X02	18.4765	0.0008	15.6322	0.0007	38.463	0.002	0.84606	0.00000	2.08171	0.00001	1667	18.31
**Steen J.**	Brawn249aX06	18.4671	0.0009	15.6381	0.0008	38.439	0.002	0.84681	0.00000	2.08147	0.00001	1668	18.30

*Copy after.

†Attribute to.

**Fig. 1.**
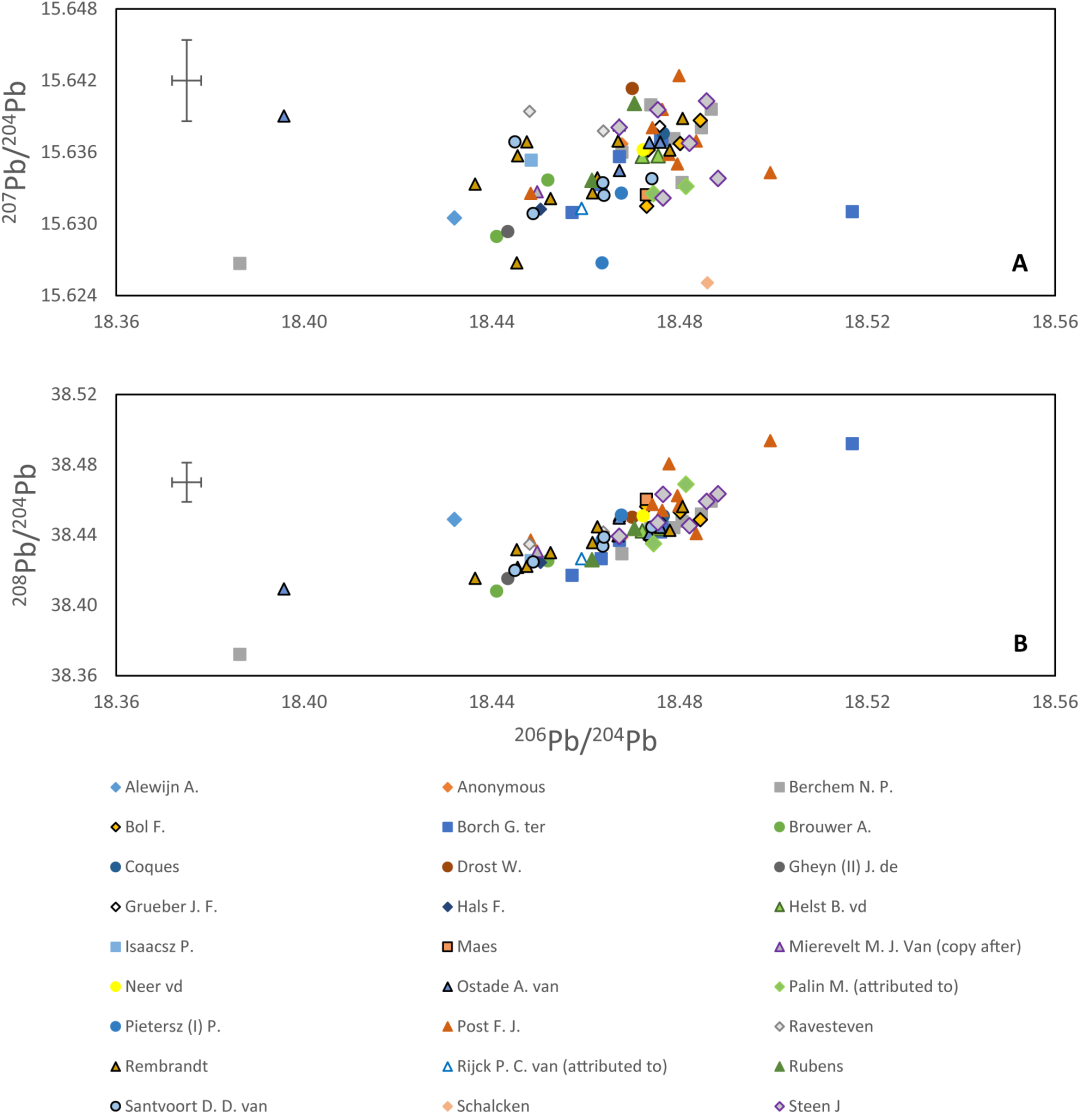
Lead isotope ratios. Three lead isotope figures reporting (**A**) ^207^Pb/^204^Pb versus ^206^Pb/^204^Pb and (**B**) ^208^Pb/^204^Pb versus ^206^Pb/^204^Pb for the studied lead white samples from this work by artist. The cross in the top left indicates the long-term reproducibility of the method.

The Pb isotope data are presented in bivariant diagrams, ^206^Pb/^204^Pb versus ^207^Pb/^204^Pb and ^206^Pb/^204^Pb versus ^208^Pb/^204^Pb (Fig. 1). The data demonstrate that there is significant variation in the lead source used in the 17th century to produce lead white, but there is no visible systematic distinction between samples produced by specific artists. Figure 1 (A and B) shows four points that are visibly distinct from the rest of the samples. These four samples failed the Grubb’s test, used to detect outliers, and therefore, they are considered outliers. The test was applied assuming a normal distribution of lead isotope ratios as the asymmetry, and kurtosis of the dataset was calculated to be less than 2. The four samples have at least one outlier for ^206^Pb/^204^Pb or ^208^Pb/^204^Pb ratio (more detailed explanation can be found in the “Outliers” section).

### Lead isotope ratios versus time

**Fig. 2.**
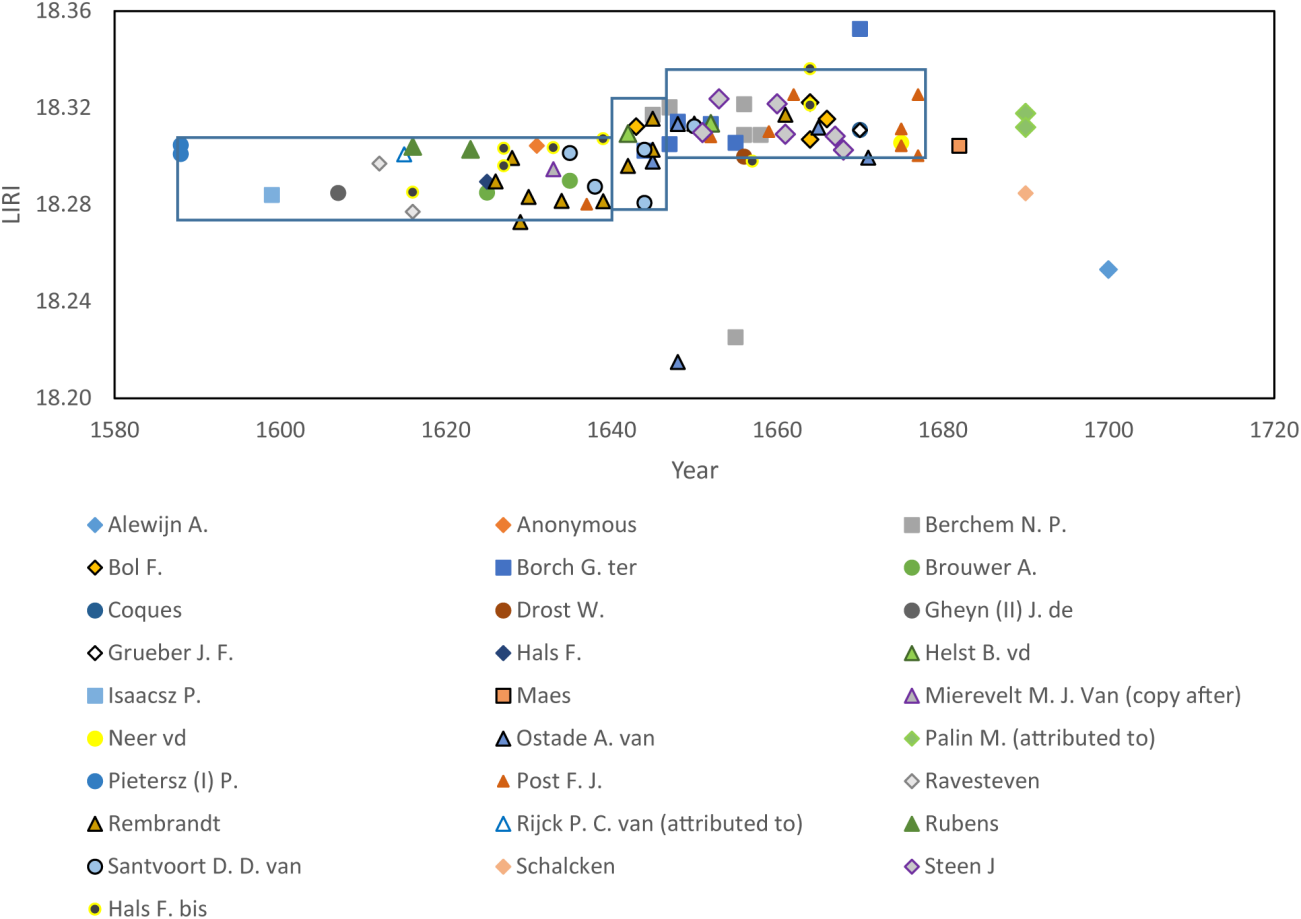
LIRI values against time. For the studied lead white, samples were divided in three clusters that cover the time periods 1588–1642, 1642–1647, and 1647–1680. The periods 1588–1642 and 1647–1680 record limited isotopic variation, indicating relatively stable lead supplies. Period 1642–1647 represents a transition of lead isotope ratios of lead white. The data include the results obtained for Frans Hals paintings (Hals F. bis) from Tummers *et al*. (*29*). The error on the LIRI is smaller than the marker.

The advantage of examining Pb isotope data on bivariant isotope ratio diagrams is that any mixing between two lead sources results in a straight line relationship. The disadvantage is that each diagram omits several degrees of freedom, recorded by time-integrated decay of the three parent-daughter isotope systems (U^235, 238^/Pb and Th^232^/Pb). Moreover, comparison of lead isotope ratios against time is more difficult, as only one isotopic ratio can be studied. Therefore, to include information from all the independent Pb isotope variables simultaneously and allow easy consideration of isotopic variations with time, the lead isotope ratios were treated following a method described by Keisch and Callahan (*5*). This approach computes a single value from all the ^20*x*^Pb/^204^Pb isotope ratios in the lead isotope ratio index (LIRI; see Materials and Methods and the Supplementary Materials). Although undoubtedly a simplification of the entire variation of the data, LIRI contains the main information recorded by all the isotope ratios, and values can be plotted against time (Fig. 2). The LIRI data show two main data clusters, 1588–1642 and 1648–1680, with a transition in the period 1642–1647. More details of the statistical significance of the differences between these two clusters is discussed in Materials and Methods. These time periods are indicated by three boxes in Fig. 2. Only three samples, already identified as potential outlier, fall outside the three main groupings identified in the period 1588–1680.

The period between 1588 and 1642 is characterized by LIRI values between 18.271 and 18.304. Although the dataset only includes two samples from the end of the 16th century, the LIRI values of these two samples are in accordance with 17th century Dutch paintings before the 1642 transition. This indicates the existence of a lead supply chain used to produce lead white that was already functional at the end of the 1500s and remained constant for at least four decades.

The 5 years after 1642 show a transition to higher LIRI values that reached an equilibrium in the period 1647–1680. It thus took around 5 years to stabilize to a new stable isotopic composition (1642–1647), which indicates a change in the source of lead supplied for the production of lead white. The timing of the observed isotopic transition coincides with three major socioeconomic events in Europe that would have had a major influence on the lead supply chain: (i) a constant increase in the demand for lead in everyday life and warfare in the 17th century. England was the major lead producer and underwent an expansion in production leading to the exhaustion of parts of some mines and the opening of new ones. These developments likely caused changes in the average lead isotope ratios of the product added to the market (*27*, *28*). (ii) The Eighty Years’ War ended in continental Europe in 1648 with the treaty of Munster, when the Dutch Republic was recognized as an independent state. Both the peace and the independence of the Dutch Republic would have led to new trading agreements and therefore to potential changes in lead supplies. (iii) The English Civil War, 1642–1651, temporarily altered the English dominance in the lead production and trading market (*28*). The 1642–1647 transition in the lead isotope ratios recoded in the LIRI values indicates, however, that the source of lead did not change immediately. This is logical because even if a new supply chain was established, resources held by suppliers would take time to be consumed, and artists would have supplies stored in their studios. The duration of the isotopic transition recorded in the LIRI values indicates that existing resources were gradually used and potentially mixed with new lead.

In the period 1647–1680, the LIRI values are between 18.297 and 18.326, with a similar degree of variation as observed for lead white used before 1642. This is an indication of a stabilization of lead supplies. Samples from paintings made after 1680 appear to be isotopically more variable, but the dataset from this period is limited. Although based on a more limited number of samples, the higher variation of isotopic values in this period could be related to the rising tension and wars associated with the Third Anglo-Dutch War (1672–1674) and the Franco-Dutch War (1672–1678). More samples and more historical documentation are needed on the trading history and mine exploitation to better characterize and understand the cause of the isotopic composition of lead white in this period.

**Fig. 3.**
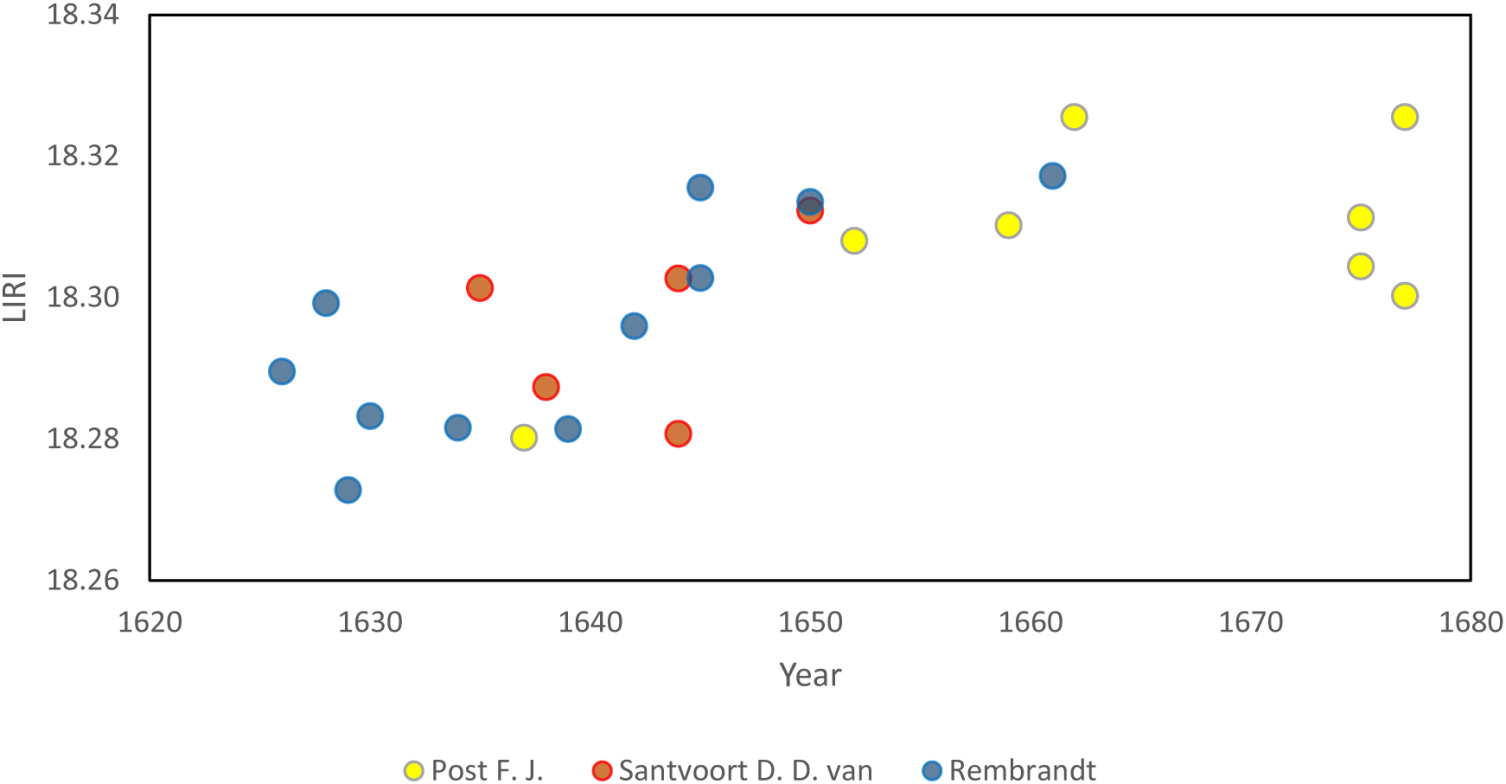
LIRI values against time. LIRI values against time for lead white from three artists who were active before and after 1645.

On the basis of the observed isotopic differences of the paintings, the LIRI values can be used to differentiate between early and late works of an individual artist. Figure 3 presents the LIRI values against time for paintings from individual artists active for many decades around the middle of the century. Paintings by F. J. Post, D. D. van Santvoort, and Rembrandt record different isotopic compositions in their early and late works. The observed temporal control in lead isotope ratios will potentially be useful for art historians as they examine the time scale for the development of an artist’s oeuvre. The key observations are that it appears possible to determine the time period of production of a Dutch painting and constrain it to the intervals outlined in Fig. 2. Unfortunately, lead isotope ratios do not have the resolution to distinguish between artists who were active in the same time period.

**Fig. 4.**
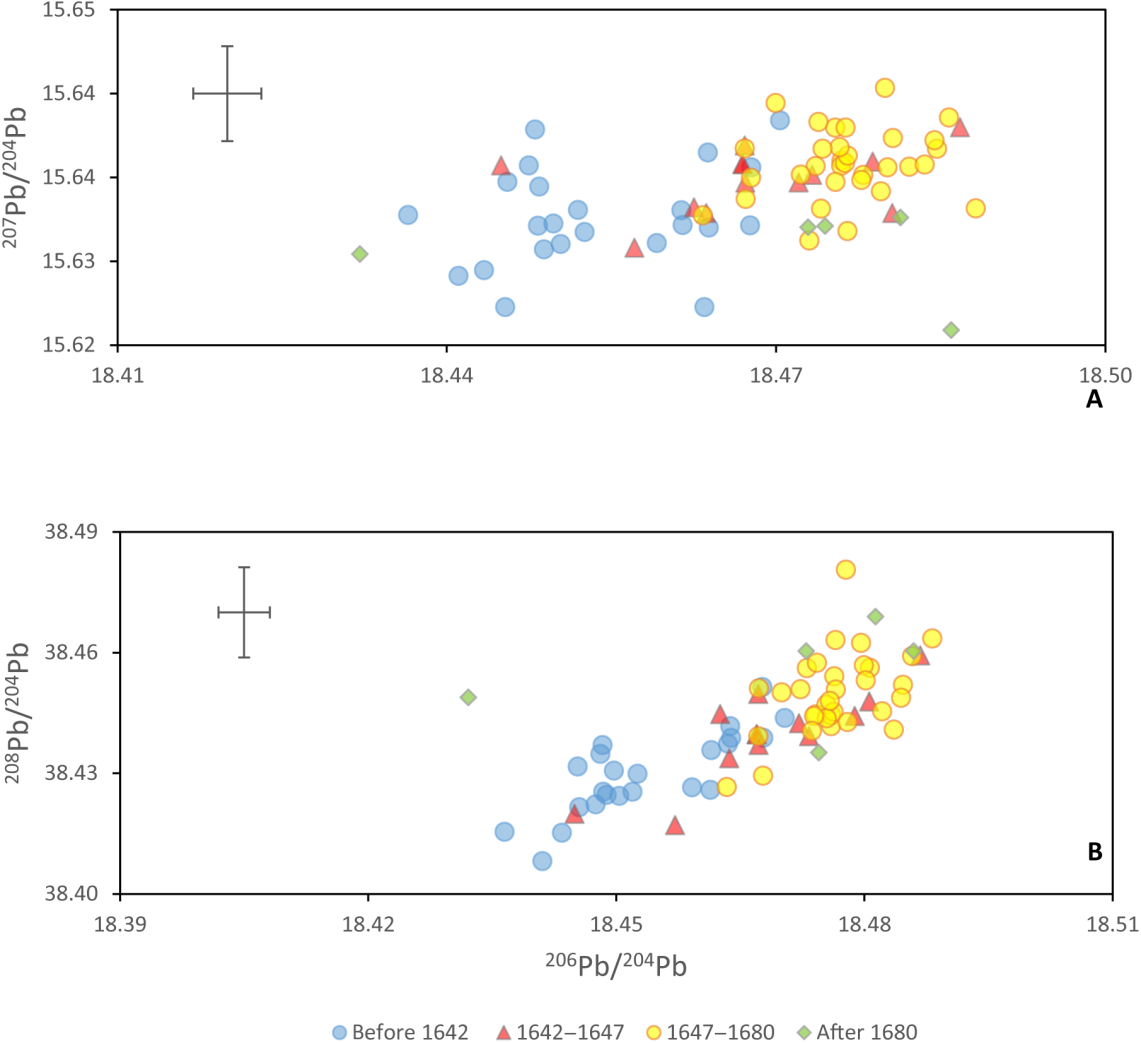
Lead isotope ratios in time. Three lead isotope figures reporting (**A**) ^207^Pb/^204^Pb versus ^206^Pb/^204^Pb and (**B**) ^208^Pb/^204^Pb versus ^206^Pb/^204^Pb for the studied lead white samples divided in temporal clusters identified used the LIRI (outliers are excluded from the diagrams). The cross in the top left corner indicates the long-term reproducibility of the method.

The temporal changes deduced from the LIRI data are used to reanalyze the ^206^Pb/^204^Pb, ^207^Pb/^204^Pb, and ^208^Pb/^204^Pb isotope ratios (Fig. 4). When the samples are grouped on the basis of temporal variation defined by the LIRI values, it is possible to distinguish paintings made in different periods of the 17th century. Paintings produced before 1642 have lower ^208^Pb/^206^Pb, ^207^Pb/^206^Pb, and ^206^Pb/^204^Pb compared to the paintings made between 1647 and 1680, while the paintings belonging to the transition period, 1642–1647, have intermediate values. The figure also establishes that the isotopic compositions of samples taken from paintings dated after 1680 are poorly constrained but indicate greater variation.

This approach establishes that LIRI proved to be a useful method to study lead isotope ratios of lead white, allowing identification of temporal clusters that can be used to better interpret the isotopic data of paintings and frame them in time. Moreover, this method also allows a comparison between the data in this study with older data. Figure 2 includes the data from Frans Hals by Tummers *et al*. (*29*) (Hals F. bis in the legend), and it is possible to see that the isotopic composition of lead white of these samples is consistent with the trend identified for 17th century Dutch paintings.

### Variation of lead isotope ratios within and between paintings

**Fig. 5.**
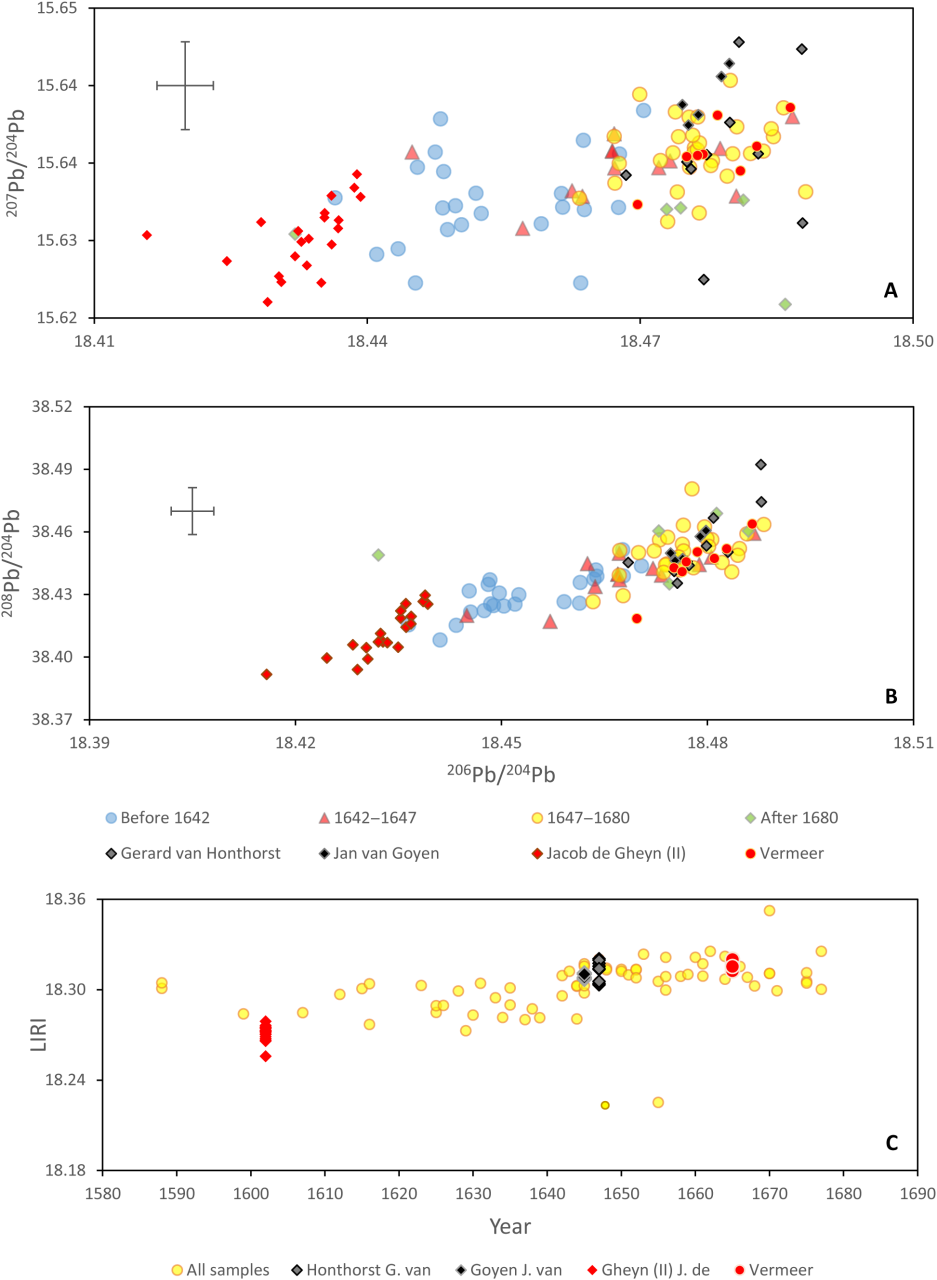
LIRI and heterogeneity of lead isotope ratios of lead white. Three lead isotope figures reporting (**A**) ^207^Pb/^204^Pb versus ^206^Pb/^204^Pb and (**B**) ^208^Pb/^204^Pb versus ^206^Pb/^204^Pb for the studied lead white samples divided in temporal clusters identified used the LIRI (outliers are excluded from the diagrams). **(C**) LIRI values of the same samples. Lead isotope heterogeneity of lead white within individual paintings presented per artist. The cross in the top left corner indicates the long-term reproducibility of the method.

To fully comprehend the significance of the observed isotopic variation in 17th century Dutch paintings, it is important to address the isotopic heterogeneity present within a single painting (*6*). This question is particularly important, as lead white is used in multiple ways in the build-up of a painting (*12*, *23*, *24*). Examinations of 17th century Dutch paintings indicate that most artists used different quality lead whites in the same painting. Although there are some variations, in general the highest quality with a finer grain size and greater purity was used for the highlights, whereas a less pure pigment with a more heterogeneous grain size was often used for other paint and ground layers or as a siccative added to the oil. D’Imporzano *et al*. (*6*) found that lead isotope values within individual paintings vary by up to four times the analytical uncertainty (2 SD) in ^206^Pb/^204^Pb, equal to a 10th of the variation of Dutch paintings in this study (44.5, 2 SD; Fig. 5, A and B).

Figure 5C reports intrapainting lead isotope ratio variation as LIRI, plotted against the dataset created for this study. The figure indicates that isotopic variance of lead white within individual paintings is within the variance recorded during the relevant time periods and hence does not affect the overall temporal variations recorded in the 17th century database. The lead isotope composition of lead white used in 17th century Dutch paintings can therefore be used to constrain the period in which an individual painting was created. The exceptions are for paintings created between 1642 and 1647, when the source of lead changed, and after 1680, the period for which the database is currently too limited.

### Outliers

The outliers samples are from the following paintings (all Rijksmuseum): *The Cattle Ferry*, SK-A-31 by N. P. Berchem c. 1655; *Aletta Pancras (1649–1707) Wife of François de Vicq*, SK-A-2418 by G. ter Borch, signed and dated 1670; *The Painter’s Studio*, SK-A-298 by A. van Ostade, c. 1647–1650; and *Brazilian Village*, SK-A-4272 by F. J. Post, 1675–1680. The Nicolaes Berchem painting was produced around 1655. It is known that the artist traveled and lived in Italy between 1651 and 1655, after which he returned to Haarlem, The Netherlands. This suggests that the lead white could be of Italian origin. This hypothesis, however, is not supported by the isotopic data. Lead isotope data for lead white used in Italy, for example, analyses of 16th century lead white cones exported from Venice, record limited variation (18.37 to 18.38, 15.67 to 15.68, and 38.52 to 38.54 for ^206^Pb/^204^Pb, ^207^Pb/^204^Pb, and ^208^Pb/^204^Pb, respectively) (*9*), with ratios comparable to literature data from 14th to 18th century Italian paintings (*6*–*8*). These isotopic compositions are markedly different from those from Berchem’s painting. The lead white has distinctly lower ^207^Pb/^204^Pb and ^208^Pb/^204^Pb ratios that are inconsistent with those associated with both Dutch or Italian lead whites or a mixture of the two. The most likely explanation appears to be that the painting was made using lead white acquired from a local and uncharacterized source during the artist’s return to The Netherlands from Italy.

The Adriaen van Ostade (SK-A-298, c. 1647–1650) painting was produced while the artist was in Haarlem, the city where he lived from 1634 until his death in 1684. The reason behind the difference in lead isotope composition of this sample compared to the rest of the samples analyzed for this work is unclear.

Two more outliers are the samples from Frans Post’s painting (SK-A-4272, c. 1675–1680) and that of Gerard ter Borch (SK-A-2418, 1670). The two paintings were produced by the artists when working in The Netherlands. Ter Borch was in Deventer in 1670, and Post was in Haarlem. These two samples come from works that are dated after 1670, a period that remains poorly characterized for lead isotope ratios. More information should be acquired about the isotopic composition of lead whites in this period to better understand the implication of these isotopic compositions.

### Paintings produced outside The Netherlands

Six paintings were produced outside The Netherlands, with five having Pb isotope ratios comparable to the lead white used in the low countries during the 17th century: *Godard van Reede (1588–1648), Lord of Nederhorst. Delegate of the Province of Utrecht at the Peace Conference at Münster (1646–48)*, SK-A-3842, G. ter Borch, 1647, Munster; *View of the Island of Itamaracá, Brazil*, SK-A-4271, F. J. Post, 1637, Brazil; *Still Life*, SK-A-2564, J. F. Grueber, c. 1670, Stuttgart; *Portrait of Rycklof van Goens, Governor-General*, SK-A-3766, and *Portrait of Cornelis Speelman, Governor-General of the Dutch East Indies*, SK-A-3767, both attributed to M. Palin, c. 1690 Jakarta; and *Cimon and Pero*, SK-C-1802, W. Drost, 1655–1659, Italy.

Paintings by Borch (1647) and Grueber (1670) were produced in the western part of Germany, Munster and Stuttgart, respectively. It is likely that Dutch lead white was used because of the proximity of the two countries and the leading position of The Netherlands as lead white producer. The painting of Post, dated 1637 in the database of Rijksmuseum, Amsterdam, is purportedly from the beginning of his stay in Brazil, where he resided from 1636 to 1644. Other studies, however, proposed that this painting was produced upon the artist’s return to The Netherlands (*30*, *31*). The lead isotope data demonstrate that the lead white used by Post is identical to that present in 17th century Dutch paintings. In particular, the LIRI indicates that the lead white is similar to that used in The Netherlands in the first half of the 1600s. At this time, The Netherlands was the major international producer of lead white, but there are no specific written records of trading lead white to Brazil. The isotopic data therefore support three viable scenarios: (i) The painting was produced by Post in the early part of his stay in Brazil and that the artist brought Dutch lead white with him; (ii) the painting was produced in Brazil with Dutch lead white imported from The Netherlands and South America; and (iii) the painting was made by the artist in The Netherlands, and according to the LIRI, it was painted shortly after his return in 1644. Further historical research is required to clarify which scenario is correct, as the lead isotope data are only able to resolve the painting was most probably produced before 1647.

The paintings attributed to Palin (1690), who was active only in Jakarta, also have isotopic values consistent with Dutch lead white. Most of Indonesia is formed by recent volcanism and hence is characterized by lead isotope ratios distinct from Europe (*32*, *33*). This implies that either Dutch lead white was traded to this part of the world during the 17th century or potentially that the attribution is incorrect and that the painting was made in The Netherlands by another artist. Further art historical research is required to resolve these possibilities.

An interesting case study is provided by the painting by W. Drost. The artist, who was a pupil of Rembrandt, was active as an independent artist in Amsterdam in the period 1652–1654 and died in Venice in 1659. On the basis of stylistic characteristics, the painting entitled *Cimon en Pero* (SK-C-1802) was thought to derive from his Italian period and to have been painted in Venice c. 1655–1657 (Table 1). The lead isotope values of lead white from this painting are consistent with 17th century Dutch lead white, in particular the years around 1650 while the artist was active in Rembrandt’s studio and setting up his own studio in Amsterdam. An Amsterdam provenance is further supported when the data are compared with samples from other paintings from Rembrandt’s workshop, for example, *The Holy Family at Night* (SK-A-4119), which was painted in the period c. 1642–1648. This lead white has lead isotope ratios comparable to Drost‘s *Cimon and Pero* (Table 1). It is unlikely that the artist brought Dutch lead white with him to Italy, as the material was readily available in Venice and of high quality. The observed Italian influences in the painting can be explained by the numerous Italian works that were in Amsterdam at the time. Moreover, Drost made a preparatory drawing for this painting, which dates from his Amsterdam period. Integrating this information with the lead isotope data strongly suggests that the painting was produced by the artist in Amsterdam before his departure to Italy in 1655.

## DISCUSSION

The lead isotope analysis of 77 dated Dutch paintings from 27 artists represents a unique database of the lead whites used in The Netherlands in the 17th century. The lead isotope compositions record a clear time dependence that is best visualized when plotting LIRI against time, but notably, all lead isotope ratios, except ^207^ Pb/^204^Pb, record a significant temporal change. There is a shift in lead isotope compositions during the period 1642–1647, and a second change is indicated after 1680.

The changes in lead isotope ratios in lead white are directly connected to historical events. The period 1642–1647 coincides with the English Civil War, when the demand for lead increased and lead production in England, the major European producer, was significantly hampered, resulting in changes in the international supply of lead. The second temporal change after the 1670s was associated with the rising tension and conflicts that took place between the English, Dutch (Third Anglo-Dutch War, 1672–1674), and French (Franco-Dutch War, 1672–1678). These conflicts undoubtedly caused alteration in the supply routes of lead. The wars also led to a huge demand for lead to produce armaments and ammunition, and therefore, less lead was available for other purposes. The possibility cannot be excluded that this situation led to the use of more local lead sources and recycling of lead with different compositions for lead white production. These observations may in part explain two of the sample outliers, the paintings by ter Borch (SK-A298, 1670) and Post (SK-A-4272, 1675) produced during the 1670s. More research is needed to investigate possible variations in lead trade and usage at the end of the 17th century.

The identification of time-dependent variation in lead isotope ratios of lead white raises the possibility of distinguishing early and late works by individual artists (Fig. 3). Moreover, the establishment of a lead isotope database (now limited to the Dutch 17th century with the possibility for extension to other periods) provides art historians a unique tool to help expand knowledge of the provenance of material used by artists and hence the dates of production of disputed works, in this case Dutch 17th century paintings. A significant observation is that some paintings produced outside The Netherlands by Dutch artists were painted using Dutch lead white, supporting the notion of the Dutch as world leaders in the production and trade of lead white. The potential power of the isotopic method is emphasized by analysis of a painting by Drost that was previously ascribed to the last years of his life during his stay in Italy. Lead isotope analysis clearly demonstrates not only that the painting was made using Dutch lead white (isotopically different from Italian lead white) but also coupled with historical research, it was possible to date the painting to around 1650, when the artist was active in Amsterdam.

Further study of 17th century Dutch paintings is required to expand the database and better characterize the beginning and end of the century. Furthermore, the temporal changes observed in Dutch paintings raise the question of how socioeconomic events influenced lead trade in other parts of Europe and hence caused changes in lead isotope ratios in lead white. On the basis of the differences in lead production and trade across Europe (*27*, *28*), different temporal variations in lead isotopes are to be expected. The lead isotope signature within The Netherlands is generally characteristic of a specific part of the 17th century. Individual artists active at the same time cannot be distinguished based on lead isotope analysis. Further systematic studies appear warranted to evaluate the full power of the technique for the cultural heritage field in providing more information on the production and trade of pigments, travel of the artists, and, in some cases, authentication-attribution of artworks. Further investigation of historical documents relevant to lead trade and lead white production will be necessary to obtain a full regional understanding of the cause of lead isotope variation across Europe.

## MATERIALS AND METHODS

### Samples

The lead white paint samples were chosen according the following criteria:

1) Samples were taken from existing cross sections.

2) The time of painting production was known to a maximum uncertainty of ±2.5 years (e.g., 1650–1655).

3) The artists were active in The Netherlands, except for selected case studies.

The location of production for each painting was researched using data from the Rijksbureau voor Kunsthistorische Documentatie (RKD)–Netherlands Institute for Art History website (https://rkd.nl/en/), which combines the year of production and the location of activity of the artist. In this way, it was possible to determine which paintings were painted outside of The Netherlands. Six paintings all from the Rijksmuseum, Amsterdam, produced outside The Netherlands were chosen to provide further insights into the habit of artists’ practices during their travels, i.e., to establish whether artists transported their own pigment materials with them or used local supplies.

### Method

Following the procedure described by D’Imporzano *et al*. (*14*), the lead white pigment samples were obtained from cross sections using a microscalpel. This method allows sampling individual paint layers of a cross section between 10 and 50 μm in width. Samples were placed in a precleaned 1.5-ml centrifuge tube and dissolved in 1 ml of 2 M HNO_3_. The solutions were then transferred to precleaned 7-ml Teflon beakers, dried down, and redissolved in 0.7 M HBr (0.2 ml). The Pb fraction was separated from the matrix by liquid chromatography using AG 1-X8 anion exchange resin (analytical grade, 200 to 400 mesh, chloride form). The concentration of the Pb fraction was determined with inductively coupled plasma mass spectrometry (ICPMS). A 2-ml 1% HNO_3_ solution containing 100 ng of Pb (50 parts per billion) was prepared for each sample. The solutions were analyzed using a Thermo Fisher multicollector ICPMS using a sample-standard bracketing (SSB) method. A NBS981 lead solution, two in-house internal standards, and a blank were analyzed to monitor data quality for each batch of 18 analyzes. The blank solutions, prepared following the same procedure as the samples, were analyzed by isotope dilution with a ^208^Pb spike solution of known isotopic composition. This procedure determines the precise amount of external lead introduced during sampling and sample preparation. In all cases, blank contribution was insignificant (<0.01%).

Measurements were performed using a desolvating nebulizer system, CETAC Aridus II, operating at approximately 4 to 5 liters min^−1^ of Ar sweep gas, 0.01 to 0.02 liters min^−1^ of nitrogen, and with temperature settings of 110°C for the spray chamber and 160°C for the membrane. Lead ion beams were about 0.2 V ppm^−1^ and were measured on Faraday cups equipped with 10^11^ ohm amplifiers. A gain calibration was performed on the 10^11^ ohm amplifiers once per week. The instrument was operated with a radiofrequency power of 1290 W. Faraday cup detectors were assigned to the following masses: ^201^Hg L4, ^202^Hg L3, ^204^Pb L2, ^205^Tl L1, ^206^Pb C, ^207^Pb H1, ^208^Pb H2, and ^209^Bi H3.

### Data analysis

The analytical method is designed to correct for isobaric interferences caused by the presence of ^204^Hg on the ^204^Pb by determining the abundance of the more abundant ^202^Hg isotope during the analyzes. No Hg interferences were detected during the analyses. An analysis of a sample consisted of one block of 100 cycles of 4-s integration time.

Lead isotope data were processed using the SSB method. The data were corrected for mass fractionation using an exponential law

$$\text{Rv}({}^{20x}\text{Pb}{/}^{20y}\text{Pb})=\text{Mv}({}^{20x}\text{Pb}{/}^{20y}\text{Pb})\cdot {\left(m^{20x}\text{Pb}/m^{20y}\text{Pb}\right)}^{\beta }$$ (1)

Rv(^20*x*^Pb/^20*y*^Pb) = real value of the (^20*x*^Pb/^20*y*^Pb) ratio.

Mv(^20*x*^Pb/^20*y*^Pb) = measured value of the (^20*x*^Pb/^20*y*^Pb) ratio.

*m* = mass of the specific lead isotope.

**β** = mass fractionation coefficient of (^20*x*^Pb/^20*y*P^b) ratio.

*x* and *y* = 4, 6, 7, or 8

During the SSB method, a standard solution NBS981, of known isotopic composition, is analyzed before and after each sample (Std1 and Std2). The measured values of the two standards are used to obtain the mass fractionation coefficient β (β_Std1_ and β_Std2_) using Eq. 1

$$\begin{array}{c} \beta_{\left(\text{Std}1,\text{Std}2\right)}=\text{ln}({\text{Rv}}_{\left(\text{Std}\right)}({}^{20x}\text{Pb}{/}^{20y}\text{Pb})/\\ {\text{Mv}}_{\left(\text{Std}\right)}({}^{20x}\text{Pb}{/}^{20y}\text{Pb}))/\text{ln}(m^{20x}\text{Pb}/m^{20y}\text{Pb}) \end{array}$$ (2)

The β_Std1_ and β_Std2_ are then used to correct for mass fractionation of the measured value of the sample according to the equation

$$\begin{array}{c} {\text{Rv}}_{\left(\text{sample}\right)}({}^{20x}\text{Pb}{/}^{20y}\text{Pb})=Mv_{\left(\text{sample}\right)}({}^{20x}\text{Pb}{/}^{20y}\text{Pb})\cdot \\ {\left(m^{20x}\text{Pb}/m^{20y}\text{Pb}\right)}^{\left((\beta \text{Std}1+\text{βStd}2)/2\right)} \end{array}$$ (3)

The error on an isotopic ratio was calculated using the SE obtained by the Thermo Fisher MC-ICPMS software. The error on the measurement is given as corrected 2SE (error propagation after the real value of the isotopic ratio is obtained using Eq. 3). The 2SE is obtained using the equation

$$2\text{SE}=2*\sqrt[{2}]{{\left(\frac{\text{SE}}{\text{Mv}}\right)}_{\text{Std}1}^{2}+{\left(\frac{\text{SE}}{\text{Mv}}\right)}_{\text{Sample}}^{2}+{\left(\frac{\text{SE}}{\text{Mv}}\right)}_{\text{Std}2}^{2}}*{\text{Rv}}_{\left(\text{sample}\right)}$$ (4)

The long-term precision of the method is calculated as twice the SD, 2SD, of replicates of an NBS981 standard [values from Thirlwall (*34*)] analyzed over a period of 2 years (*N* > 100). This is equal to 0.0031 for the ^206^Pb/^204^Pb, 0.0034 for the ^207^Pb/^204^Pb, 0.011 for the ^208^Pb/^204^Pb, 0.00008 for the ^207^Pb/^206^Pb, and 0.0002 for the ^208^Pb/^206^Pb.

### Lead Isotope Ratio Index

The observed higher variation in the ^206^Pb/^204^Pb ratio compared to ^207^Pb/^204^Pb and ^208^Pb/^204^Pb ratios is related to the rate U and Th decay and comparable to previous analyzes made on lead white (*5*–*8*). These variations are consistent with the isotopic variation in lead ores worldwide that record >40% variation in ^206^Pb/^204^Pb and ~6.5% in ^207^Pb/^204^Pb. Keisch and Callahan (*5*) previously identified that ^206^Pb/^204^Pb is the most powerful ratio for identifying patterns of lead isotope variation in populations of lead white. From their study of hundreds of samples from paintings from the 12th to 20th century, Keisch and Callahan (*5*) identified several clusters in a two-dimensional graph, ^207^Pb/^204^Pb versus ^206^Pb/^204^Pb. By outlining the points in each cluster, they identify that the preponderant distinguishing characteristic was the ^206^Pb/^204^Pb ratio. They also observed that the clusters were all similar in shape, essentially consisting of ellipses with the long axes inclined at about 45° from lower left to upper right. These ellipses were all distributed along a horizontal line at a ^207^Pb/^204^Pb value that averaged approximately 15.5. Therefore, the values of the ^206^Pb/^204^Pb ratios were modified by applying a correction based upon the ^207^Pb/^204^Pb ratio so as to project the former ratio onto a horizontal line at ^207^Pb/^204^Pb = 15.5 along a line parallel to the axes of the data ellipses. These transpositions yielded a set of once-modified ^206^Pb/^204^Pb ratios designated (6/4)′. A similar plot of ^208^Pb/^206^Pb versus (6/4)′ yielded an additional modification, which, in turn, was computed to twice-modified ratios designated (6/4)″ (*5*).

In this way, the LIRI was developed, and it applies a correction to ^206^Pb/^204^Pb based on ^207^Pb/^204^Pb and ^208^Pb/^206^Pb values following the empirical equation

LIRI = 35.385 + 0.4729 * (^206^Pb/^204^Pb) − 0.5519 * (^206^Pb/^204^Pb) * (^207^Pb/^206^Pb) − 8.2561 * (^208^Pb/^206^Pb)

The LIRI index gives an indication of whether a sample lies within a specific cluster of isotopic ratios and allows the integrated isotopic data to be easily plotted against time. If direct comparison of two samples is required, then it is important to analyze the data using the original lead isotope ratios, because identical LIRI values can be obtained from different raw data (*5*). More detailed reasons for use of the LIRI are given in the Supplementary Materials.

### Statistical significance test of LIRI values before and after 1642–1647

The identification of two clusters of paintings requires statistical validation. Therefore, a null hypothesis (no significant difference in the paintings present in the two groups) and *P* values (95% of confidence) were considered. The *P* values were calculated for the LIRI values of the 25 paintings dated between 1588 and 1642 and 36 paintings made in the period 1648–1680. For the calculation, Microsoft Excel software (*t* test: two sample assuming unequal variances, two tails) was used. The calculated *P* values are equal to 0.009, with *P* < 0.05 proving that the null hypothesis can be discarded, and therefore, it is possible to state that the two groups are significantly different.

## Supplementary Materials

### This PDF file includes:

Table S1 Figs. S1 to S4 References
