# Supplementary material for: Time-dependent variation of lead isotopes of lead white in 17th century Dutch paintings
Source: Sci Adv. 2021 Dec 1;7(49):eabi5905. doi: 10.1126/sciadv.abi5905 (PMC8635429; doi:10.1126/sciadv.abi5905)
Supplement: Supplementary file 1 — Table S1 Figs. S1 to S4 References [file sciadv.abi5905_sm.pdf]

Science Advances
AAAS

Supplementary Materials for

Time-dependent variation of lead isotopes of lead white in 17th century Dutch paintings

P. D'Imporzano*, K. Keune, J. M. Koornneef, E. Hermens, P. Noble, A. L. S. Vandivere, G. R. Davies

*Corresponding author. Email: pdimpo@hotmail.com

Published 1 December 2021, *Sci. Adv.* **7**, eabi5905 (2021)
DOI: 10.1126/sciadv.abi5905

This PDF file includes:

Table S1
Figs. S1 to S4
References

Artist	Museum ID	Museum	Title
Alewijn A.	SK-A-1726	Rijksmuseum	Marine Pageant on the Y off Amsterdam
anonymous	SK-A-1312	Rijksmuseum	Portrait of Gerrit Ottsz Hinlopen
Berchem N. P.	SK-A-27	Rijksmuseum	The City Wall of Haarlem in the Winter
Berchem N. P.	SK-A-32	Rijksmuseum	Ruth and Boas
Berchem N. P.	SK-A-28	Rijksmuseum	On the Ice near a Town
Berchem N. P.	SK-A-31	Rijksmuseum	The Cattle Ferry
Berchem N. P.	SK-A-29	Rijksmuseum	The Three Droves
Berchem N. P.	SK-A-30	Rijksmuseum	The Cattle Herd
Berchem N. P.	SK-A-680	Rijksmuseum	Ruins in Italy
Bol F.	SK-A-714	Rijksmuseum	Portrait of an Old Lady, Possibly Elisabeth Bas
Bol F.	SK-A-613	Rijksmuseum	Consul Titus Manlius Torquatus Orders the Beheading of his Son
Bol F.	SK-A-614	Rijksmuseum	Aeneas at the Court of Latinus
Bol F.	SK-A-45	Rijksmuseum	Caritas: Joanna de Geer (1629-1691) with her Children Cecilia Trip (1660-1728) and Laurens Trip (b. 1662)
Borch G. ter	SK-A-1784	Rijksmuseum	Gerard Abrahamsz van der Schalcke (1609-67). Haarlem Cloth Merchant
Borch G. ter	SK-A-3842	Rijksmuseum	Godard van Reede (1588-1648), Lord of Nederhorst. Delegate of the Province of Utrecht at the Peace Conference at Münster (1646-48)
Borch G. ter	SK-A-1786	Rijksmuseum	Helena van der Schalcke
Borch G. ter	SK-A-4039	Rijksmuseum	Woman at a Mirror
Borch G. ter	SK-A-4038	Rijksmuseum	Seated Girl in Peasant Costume
Borch G. ter	SK-A-2417	Rijksmuseum	François de Vicq, Burgomaster of Amsterdam for several Terms from 1697on
Brouwer A.	MH919 2a	Mauritshuis	Fighting Peasants
Brouwer A.	MH067x2	Mauritshuis	A Fat Man
Coques	238X18 9/01MW	Mauritshuis	Interior with Figures in a Picture Gallery
Drost W.	SK-C-1802	Rijksmuseum	Cimon and Pero
Gheyn (II) J. de	SK-A-2395	Rijksmuseum	Venus and Amor
Grueber J. F.	SK-A-2564	Rijksmuseum	Still life
Hals F.	459*10a06	Mauritshuis	Portrait of Jacob Olycan (1596-1638)
Helst B. vd	SK-A-147	Rijksmuseum	Portrait of Gerard Andriesz Bicker
Helst B. vd	SK-A-142	Rijksmuseum	Mary Stuart, Princess of Orange, as Widow of William II

Isaacsz P.	SK-C-455	Rijksmuseum	The Company of Captain Gillis Jansz Valckenier and Lieutenant Pieter Jacobsz Bas, Amsterdam, 1599
Maes	MH0718X01	Mauritshuis	Portrait of Catharina Dierquens (1664-1715)
Mierevelt M. J. Van*	SK-C-1481	Rijksmuseum	Portrait of Gustav II Adolf (1594-1632), King of Sweden
Neer vd	862X01 2011 SM	Mauritshuis	nterior with a Woman Washing her Hands
Ostade A. van	SK-A-4093	Rijksmuseum	Landscape with an old oak
Ostade A. van	SK-A-300	Rijksmuseum	The Quacksalver
Ostade A. van	SK-A-3281	Rijksmuseum	Portrait of a Collector, possibly Constantijn Sennepart (1625-1703)
Ostade A. van	SK-A-299	Rijksmuseum	Travellers at Rest
Ostade A. van	SK-A-298	Rijksmuseum	The Painter's Studio
Palin M.**	SK-A-3766	Rijksmuseum	Portrait of Rycklof van Goens, Governor-General
Palin M.**	SK-A-3767	Rijksmuseum	Portrait of Cornelis Speelman, Governor-General of the Dutch East Indies
Pietersz (I) P.	SK-A-3864	Rijksmuseum	Portrait of Mattheus Augustijnsz Steyn, Councilor in the College of the Admiralty of the Northern Quarter in Dokkum
Pietersz (I) P.	SK-A-3865	Rijksmuseum	Portrait of Dirckje Tymansdr Gael, called van der Graft, Wife of Mattheus Augustijnsz Steyn
Post F. J.	SK-A-4271	Rijksmuseum	View of the Island of Itamaracá, Brazil
Post F. J.	SK-A-3224	Rijksmuseum	Landscape in Brazil
Post F. J.	SK-A-1486	Rijksmuseum	Brazilian landscape with the village of Igaraçú. To the left the church of Sts Cosmas and Damian
Post F. J.	SK-A-742	Rijksmuseum	View of Olinda, Brazil
Post F. J.	SK-A-2333	Rijksmuseum	Brazilian Landscape
Post F. J.	SK-A-2334	Rijksmuseum	Landscape on the Rio Senhor de Engenho, Brazil
Post F. J.	SK-A-4272	Rijksmuseum	Brazilian Village
Post F. J.	SK-A-4273	Rijksmuseum	Church Building in Brazil
Ravesteven	421-2-CE	Mauritshuis	Portrait of an Officer
Ravesteven	142/08/98	Mauritshuis	Portrait of an Officer
Rembrandt	SK-A-4717	Rijksmuseum	Tobit and Anna with the Kid
Rembrandt	SK-A-4691	Rijksmuseum	Self-portrait
Rembrandt	148/3/98	Mauritshuis	Portrait of Rembrandt (1606-1669) with a Gorget
Rembrandt	MH0565x08	Mauritshuis	ronie' of an Old Man
Rembrandt	SK-A-4833	Rijksmuseum	Portrait of Haesje Jacobsdr van Cleyburg

Rembrandt	SK-A-1935	Rijksmuseum	Landscape with a Stone Bridge
Rembrandt	SK-A-91	Rijksmuseum	Salome ontvangt het hoofd van Johannes de Doper
Rembrandt	SK-A-4119	Rijksmuseum	The Holy Family at Night
Rembrandt	SK-A-4119	Rijksmuseum	The Holy Family at Night
Rembrandt	560-3b-CE98	Mauritshuis	Study of an Old Man
Rembrandt	SK-A-4050	Rijksmuseum	Self-portrait as the Apostle Paul
Rijck P. C. van**	SK-A-868	Rijksmuseum	Kitchen Scene with the Parable of the Rich Man and Poor Lazarus
Rubens	252/1098	Mauritshuis	Portrait of Michael Ophovius (1570-1637)
Rubens	926*05'5BS	Mauritshuis	Modello' for the Ascension of the Virgin
Santvoort D. D. van	SK-A-365	Rijksmuseum	Portrait of the Family of Dirck Bas Jacobsz, Burgomaster of Amsterdam
Santvoort D. D. van	SK-A-1318	Rijksmuseum	Agatha Geelvinck (1617-38). First wife of Frederik Dircksz Alewijn, Dirck Dircksz
Santvoort D. D. van	SK-A-1310	Rijksmuseum	Portrait of Martinus Alewijn
Santvoort D. D. van	SK-A-1311	Rijksmuseum	Portrait of Clara Alewijn
Santvoort D. D. van	SK-A-1623	Rijksmuseum	Portrait of Machteld Bas (- 1681)
Schalcken	160X2 1.11CP	Mauritshuis	A Useless Moral Lesson
Steen J	MH0165x02	Mauritshuis	The Tooth-Puller
Steen J	Brawn 056x02	Mauritshuis	n.a.
Steen J	MH0166x05	Mauritshuis	n.a.
Steen J	B143x01	Mauritshuis	n.a.
Steen J	Brawn363X02	Mauritshuis	n.a.
Steen J	Brawn249aX06	Mauritshuis	n.a.

*Table S1: Paintings sorted according to Artist. For each painting is reported: sample museum ID, Museum of provenance, and title of the painting. * copy after ** attribute to.*

Supplementary material on the use of LIRI

Various approaches were considered to visualise the variation of lead isotope ratios with time for 17th century Dutch paintings. In this work the LIRI value was chosen to assess isotopic variation with time, partly because this approach has been used by previous workers. The initial analysis was performed by evaluating each lead isotope ratio against time, as in Figure S1. The data establish that four ratios, $^{208}Pb/^{204}Pb$, $^{206}Pb/^{204}Pb$, $^{207}Pb/^{206}Pb$ and $^{208}Pb/^{206}Pb$, record recognisable change with time. Individual ratios, however, do not incorporate all the variations. For example, the isotopic composition of the 16th century samples have distinct $^{207}Pb/^{206}Pb$ ratios to samples from the start of the 17th century but are not distinct for $^{208}Pb/^{206}Pb$.

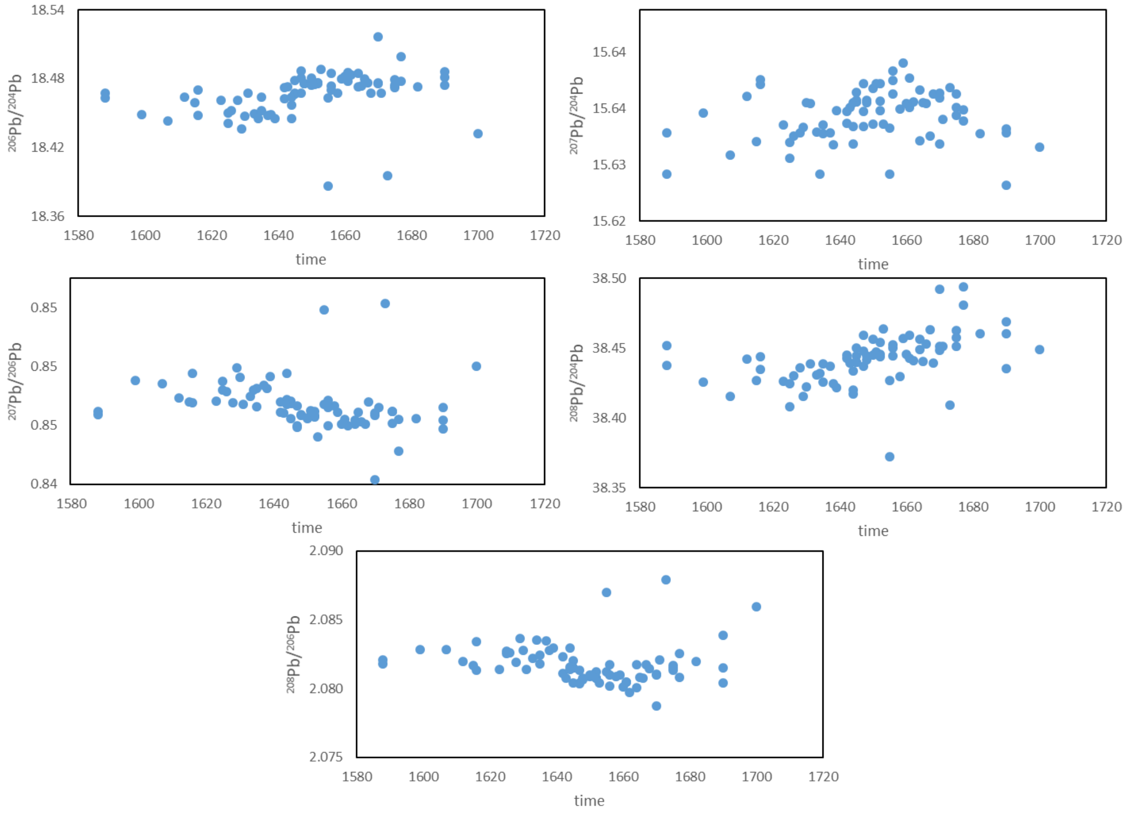

Figure S1: lead isotope ratios $^{206}Pb/^{204}Pb$, $^{207}Pb/^{206}Pb$, $^{208}Pb/^{206}Pb$, $^{207}Pb/^{204}Pb$ and $^{208}Pb/^{204}Pb$ vs time

The power of the LIRI approach is that it integrates the variation of all the lead isotope ratios in a single value that can then be presented in a 2D figure. LIRI values are calculated according to the equation:

$$\text{LIRI} = 35.385 + 0.4729 * (^{206}\text{Pb}/^{204}\text{Pb}) - 0.5519 * (^{206}\text{Pb}/^{204}\text{Pb}) * (^{207}\text{Pb}/^{206}\text{Pb}) - 8.2561 * (^{208}\text{Pb}/^{206}\text{Pb})$$

A principal component analysis (PCA) has been applied to the 5 isotope ratios obtained for each sample $^{206}Pb/^{204}Pb$, $^{207}Pb/^{204}Pb$, $^{208}Pb/^{204}Pb$, $^{207}Pb/^{206}Pb$ and $^{208}Pb/^{206}Pb$. PCA is a data analysis tool that is used in exploratory data analysis and for making predictive models.

Principal component analysis (PCA) is a multivariate technique that analyses data in which observations are described by inter-correlated quantitative dependent variables. The PCA perform a dimensionality reduction of the data to obtain lower-dimensional data while preserving as much of the data's variation as possible. The purpose of the PCA is to extract the important information from the data matrix, to obtain a set of new orthogonal variables (principal components) (35).

The first principal component produced in this study records 76% of the variance while the PC2 contains only 18%. The contribution to the variance in PC1 was given mainly by $^{206}Pb/^{204}Pb$, $^{207}Pb/^{206}Pb$, $^{208}Pb/^{206}Pb$ and $^{208}Pb/^{204}Pb$ Figure S2.

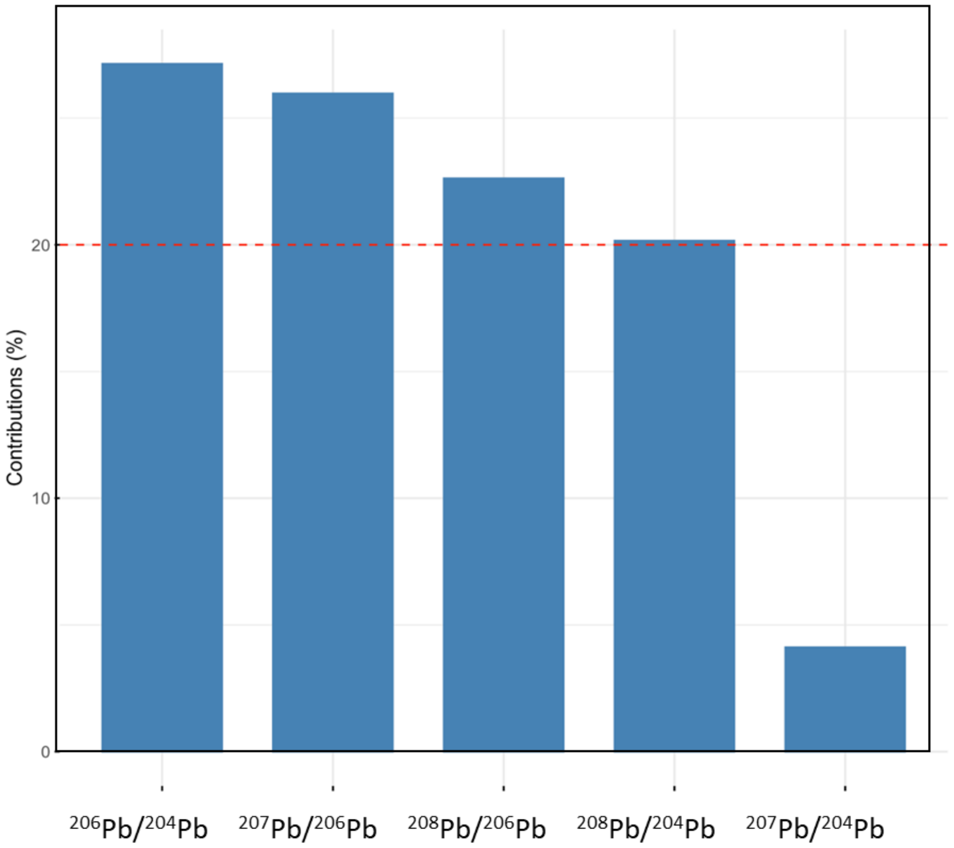

Figure S2: variables contribution on the PC1

Plotting PC2 against time do not give any information as the variance of the data on this component is low. If the PC1 is plotted against time the results it is similar to the one obtained using the LIRI, Figure S3. Both LIRI and PC1 confirms that $^{206}Pb/^{204}Pb$ contains the main information on the variation of lead isotope composition over time, however studying only the $^{206}Pb/^{204}Pb$ ratio is not sufficient as much information given by other ratios are lost.

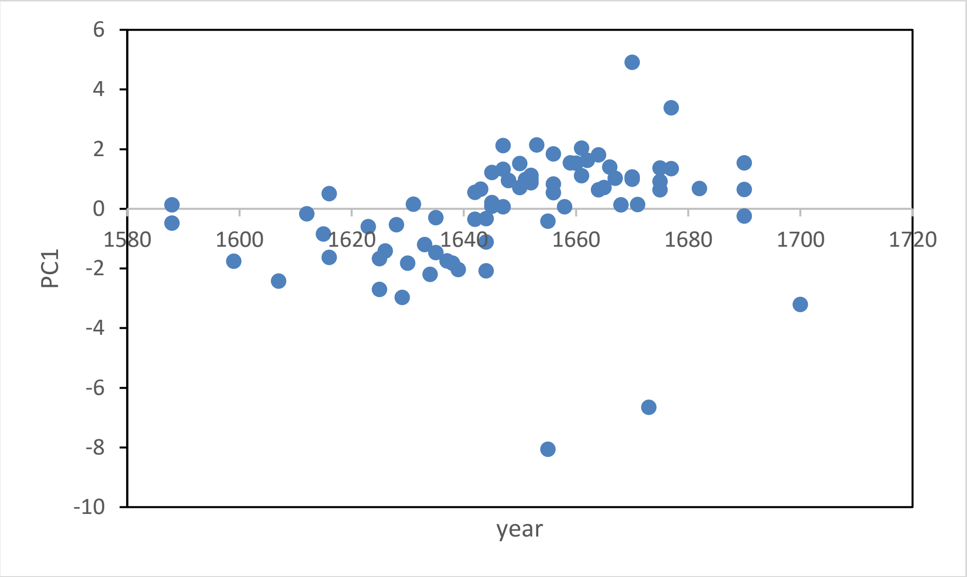

Figure S3: PC1 against time

It is interesting to note that if the LIRI is plotted against the PC1 it gives a straight line with a correlation factor of 0.9, meaning that the two transformations of the data are similar, Figure S4.

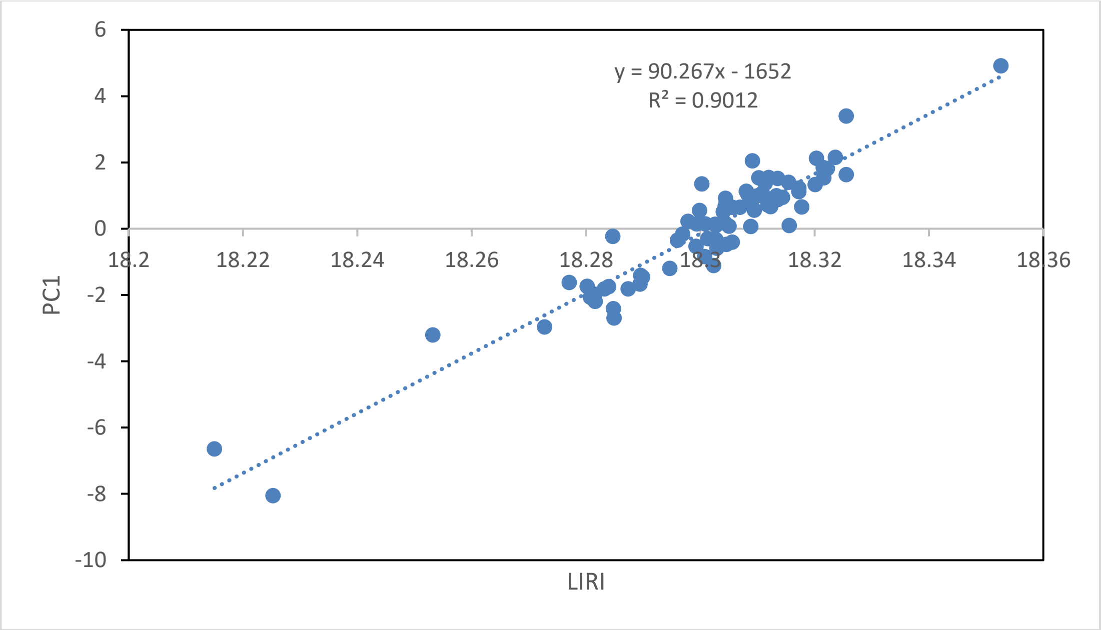

Figure S4: Correlation between LIRI and PC1

The PCA information, however, is limited to the dataset population, and for every new sample a new PCA should be calculated, making this method difficult to allow study between different datasets. By contrast, the LIRI instead is a model based on a much larger dataset, covering a much broader timeline and can be applied offline and compared directly to the results of other studies. This makes the use of LIRI values effective for the study of Pb isotope variations in painting samples.

REFERENCES AND NOTES

1. M. Pollard, C. Heron, Chapter 9 lead isotope geochemistry and the trade in metals, in *Archaeological Chemistry* (The Royal Society of Chemistry, ed. 2, 2008), pp. 302–345.

2. Z. A. Stos-Gale, N. H. Gale, Metal provenancing using isotopes and the Oxford archaeological lead isotope database (OXALID). *Archaeol. Anthropol. Sci.* **1**, 195–213 (2009).

3. J. Kershaw, S. W. Merkel, Silver recycling in the Viking Age: Theoretical and analytical approaches. *Archaeometry* **2021**, 1–18 (2021).

4. F. Albarède, A. M. Desaulty, J. Blichert-Toft, A geological perspective on the use of Pb isotopes in archaeometry. *Archaeometry* **54**, 853–867 (2012).

5. B. Keisch, R. C. Callahan, Lead isotope ratios in artists' lead white: A progress report. *Archaeometry* **18**, 181–193 (1976).

6. P. D'Imporzano, K. Batur, K. Keune, J. M. Koornneef, E. Hermens, P. Noble, K. van Zuilen, G. R. Davies, Lead isotope heterogeneity in lead white: From lead white raw pigment to canvas. *Microchem. J.* **163**, 1–10 (2020).

7. D. Fabian, G. Fortunato, Tracing white: a study of lead white pigments found in seventeenth-century paintings using high precision lead isotope abundance ratios, in *Trade in Artists' Materials: Markets and Commerce in Europe to 1700* (2010), pp. 426–443.

8. G. Fortunato, A. Ritter, D. Fabian, Old Masters' lead white pigments: Investigations of paintings from the 16th to the 17th century using high precision lead isotope abundance ratios. *Analyst* **130**, 898–906 (2005).

9. A. Roy, Artists' pigments, in *A Handbook of Their History and Characteristics* (National Gallery of Art, 67–79, 1993), vol. 2.

10. J. Olby, The basic lead carbonates. *J. Inorg. Nucl. Chem.* **28**, 2507–2512 (1966).

11. V. Gonzalez, M. Cotte, G. Wallez, A. van Loon, W. De Nolf, M. Eveno, K. Keune, P. Noble, J. Dik, Unraveling the composition of rembrandt's impasto through the identification of unusual plumbonacrite by multimodal x-ray diffraction analysis. *Angew. Chem. Int. Ed.* **58**, 5619–5622 (2019).

12. M. Stols-Witlox, L. Megens, L. Carlyle, To prepare white excellent...": Reconstructions investigating the influence of washing, grinding and decanting of stack-process lead white on pigment composition and particle size, in *The Artist's Process: Technology and Interpretation* (Archetype, 2012), pp. 112–129.

13. E. Welcomme, P. Walter, P. Bleuet, J. L. Hodeau, E. Dooryhee, P. Martinetto, M. Menu, Classification of lead white pigments using synchrotron radiation micro x-ray diffraction. *Appl. Phys.* **89**, 825–832 (2007).

14. P. D'Imporzano, K. Keune, J. M. Koornneef, E. Hermens, P. Noble, K. van Zuilen, G. R. Davies, Micro-invasive method for studying lead isotopes in paintings*. *Archaeometry* **62**, 796–809 (2020).

15. L. Hendriks, S. Kradolfer, T. Lombardo, V. Hubert, M. Küffner, N. Khandekar, I. Hajdas, H.-A. Synal, B. Hattendorf, D. Günther, Dual isotope system analysis of lead white in artworks. *Analyst* **145**, 1310-E1318 (2020).

16. J. Wadum, in *Studies in the History of Art, 55. Center for Advanced Study in the Visual Arts, Symposium Papers XXXIII,* I. J. Gaskel, M., 201–223 (1998).

17. A. K. Wheelock Jr., "St. Praxedis": New light on the early career of Vermeer. *Artibus et Historiae* **7**, 71–89 (1986).

18. A. Wallert, G. R. Davies, in *Burlington Magazine* (2021).

19. L. Beck, I. Caffy, E. Delqué-Količ, C. Moreau, J.-P. Dumoulin, M. Perron, H. Guichard, V. Jeammet, Absolute dating of lead carbonates in ancient cosmetics by radiocarbon. *Commun. Chem.* **1**, 34 (2018).

20. G. Faure, *Principles of Isotope Geology* (Wiley, ed. 2, 1986).

21. B. L. Gulson, *Lead Isotope in Mineral Exploration* (1986).

22. D. F. Sangster, P. M. Outridge, W. J. Davis, Stable lead isotope characteristics of lead ore deposits of environmental significance. *Environ. Rev.* **8**, 115–147 (2000).

23. E. Homburg, E. Homburg and J.H. de Vlieger, A victory of practice over science: The unsuccessful modernisation of the Dutch white lead industry (1780–1865). *Hist. Technol.* **13**, 33–52 (1996).

24. M. Stols-Witlox, Historical recipes for preparatory layers for oil paintings in manuals, manuscripts and handbooks in North West Europe, 1550–1900: analysis and reconstructions, PhD thesis, Amsterdam (2014).

25. B. Berrie, L. Matthew, Lead white from Venice: A whiter shade of pale, in *Studying Old Master Paintings: Technology and Practice* (2011), pp. 295–301.

26. A. van Loon, A. Vandivere, J. K. Delaney, K. A. Dooley, S. De Meyer, F. Vanmeert, V. Gonzalez, K. Janssens, E. Leonhardt, R. Haswell, S. de Groot, P. D'Imporzano, G. R. Davies, Beauty is skin deep: The skin tones of Vermeer's Girl with a Pearl Earring. *Herit. Sci.* **7**, 102 (2019).

27. I. Blanchard, *International Lead Production and Trade in the" Age of the Saigerprozess": 1460–1560* (Franz Steiner Verlag GmbH, 1995), vol. 85, pp. 167–193.

28. R. Burt, The transformation of the non-ferrous metals industries in the seventeenth and eighteenth centuries. *Econ. Hist. Rev.* **48**, 23–45 (1995).

29. A. Tummers, A. Wallert, N. de Keyser, *Burlington Magazine* (Burlington Magazine Publications Ltd., 2019), vol. 161, pp. 996–1003.

30. J. Wadum, *Albert Eckhout returns to Brasil 1644–2002: International Experts Symposium*, E. de Vries, Ed. (2002), pp. 273–279.

31. M. Roscam Abbing, *Brazilië Zien Zonder de Oceaan Over te Steken. De Wandtapijten van Johan Maurits* (Lias, 2021).

32. A. Stolz, R. Varne, G. Davies, G. Wheller, J. Foden, Magma source components in an arc-continent collision zone: The Flores-Lembata sector, Sunda arc, Indonesia. *Contrib. Mineral. Petrol.* **105**, 585–601 (1990).

33. P. Vroon, M. Van Bergen, W. White, J. Varekamp, Sr-Nd-Pb isotope systematics of the Banda Arc, Indonesia: Combined subduction and assimilation of continental material. *J. Geophys. Res. Solid Earth* **98**, 22349–22366 (1993).

34. M. F. Thirlwall, Multicollector ICP-MS analysis of Pb isotopes using a 207pb-204pb double spike demonstrates up to 400 ppm/amu systematic errors in Tl-normalization. *Chem. Geol.* **184**, 255–279 (2002).

35. H. Abdi, L. J. Williams, Principal component analysis. *Wiley Interdiscip. Rev. Comput. Stat.* **2**, 433–459 (2010).
